# Evaluation of serum CA125-Tn glycoform in peritoneal dissemination and surgical completeness of high-grade serous ovarian cancer

**DOI:** 10.1186/s13048-022-01066-1

**Published:** 2022-12-23

**Authors:** Xiayu Jin, Ming Du, Yisheng Wang, Yuefei Wang, Yingying Lu, Congjian Xu, Xiaoyan Zhang

**Affiliations:** 1 Obstetrics and Gynecology Hospital, Fudan University, 200011 Shanghai, China; 2 Shanghai Key Laboratory of Female Reproductive Endocrine Related Diseases, 200011 Shanghai, China; 3 Department of Obstetrics and Gynecology of Shanghai Medical School, Fudan University, Shanghai 200032, China

**Keywords:** Ovarian cancer, CA125, Tn antigen, Cytoreductive surgery, Peritoneal dissemination, Residual disease

## Abstract

**Background:**

Peritoneal dissemination is the predominant feature of malignant progression in ovarian cancer and is a major cause of poor surgical outcomes and clinical prognoses. Abnormal glycosylation of carbohydrate antigen 125 (CA125) may be involved in peritoneal implantation and metastasis. Here, we evaluated the clinical relevance of CA125-Tn glycoform in the assessment of high-grade serous ovarian cancer (HGSOC).

**Methods:**

A total of 72 patients diagnosed with HGSOC were included. Pre-treatment serum CA125-Tn levels were measured using an antibody-lectin enzyme-linked immunosorbent assay. The association of CA125-Tn with clinical factors was analyzed in all cases, whereas its association with peritoneal dissemination, residual disease, and progression-free survival was analyzed in stage III–IV cases.

**Results:**

Pre-treatment serum CA125-Tn levels were significantly higher in advanced-stage HGSOC patients than in early-stage patients (*P* = 0.029). In advanced-stage patients, the pre-treatment CA125-Tn level increased with an increase in Fagotti’s score (*P* = 0.004) and with the extension of peritoneal dissemination (*P* = 0.011). The pre-treatment CA125-Tn level increased with the volume of residual disease (*P* = 0.005). The association between CA125-Tn level and suboptimal surgery remained significant even after adjustment for treatment type and stage. Pre-treatment CA125-Tn levels were also related to disease recurrence.

**Conclusion:**

Serum CA125-Tn level could be a novel biomarker for peritoneal dissemination and a promising predictor of surgical completeness in ovarian cancer. Patients with lower CA125-Tn levels were more likely to have no residual disease. CA125-Tn could help surgeons to adopt optimized treatment strategies for patients with advanced ovarian cancer as a pre-treatment evaluator.

## Introduction

High-grade serous ovarian cancer (HGSOC) is the most common and aggressive type of ovarian cancer [1]. Most HGSOCs are diagnosed at an advanced stage and are the leading cause of female genitourinary cancer-related deaths [2]. Staging surgery or primary debulking surgery (PDS) combined with platinum-based chemotherapy is the standard regimen for HGSOC.

Peritoneal dissemination impairs surgical completeness and subsequently causes disease recurrence and poor prognosis in patients with advanced ovarian cancer. Approximately 20% of International Federation of Gynecology and Obstetrics (FIGO) stage I–IIA patients and 62.1% of stage IIb–IV patients experience peritoneal recurrence [3]. Small peritoneal metastases can hardly be identified using conventional computed tomography (CT) or magnetic resonance imaging and are easily ignored during surgery [4–6]. Even if peritoneal lesions can be detected, massive or complex disseminated patterns make it extremely difficult to achieve R0 resection, particularly for upper abdominal lesions and miliary lesions [7]. Residual disease (RD) after surgery is the most important risk factor for poor clinical outcomes. Patients with residuals have worse prognosis, with median survival of 29.6 months in patients with RD > 1 cm and 36.2 months in patients with RD 0.1–1 cm, which is significantly shorter than 99.1 months in R0 patients [8]. Moreover, patients with RD are prone to recurrence, with a median survival of only 12–24 months after relapse [9]. Preoperative evaluation of peritoneal dissemination is necessary to determine the R0 resection rate and survival advantage for advanced ovarian cancer.

Serum carbohydrate antigen 125 (CA125), encoded by MUC16, is the most valuable FDA-approved biomarker for ovarian cancer. CA125 may not only be a diagnostic biomarker but may also promote tumor progression in ovarian cancer [10–12]. CA125 overexpression increases colony formation and invasion in the ovarian cancer cell line SKOV3 and promotes tumor initiation and peritoneal dissemination in nude mice [13]. Strong adhesion can be detected between CA125-expressed cells and mesothelin-transfected cells, even under peritoneal fluid sheer stress [14–16]. Moreover, CA125 can specifically bind to mesothelin and contribute to peritoneal dissemination [17]. CA125 may inhibit cellular apoptosis in peritoneal fluid by mediating the epithelial–mesenchymal transition process and regulating multicellular aggregates [18–20]. A previous analysis indicated a potential relationship between CA125 and peritoneal dissemination in ovarian cancer. A high level of ascites CA125 is associated with the presence of peritoneal carcinomatosis in ovarian cancer patients [21]. However, the association between serum CA125 level and peritoneal carcinomatosis remains controversial. Serum CA125 levels may be higher or similar in ovarian cancer patients with peritoneal carcinomatosis than in those without peritoneal carcinomatosis [21, 22].

As the most abundant post-translational modification and a common regulator of protein regulation, glycosylation may mediate malignant transformation and metastasis [23–25]. CA125 is a highly glycosylated mucin. Ovarian cancer cells often exhibit a truncated O-glycophenotype of CA125. Salminen et al. found that CA125-sialyl-Tn (CA125-sTn) and CA125-MGL differentiated ovarian cancer better than the conventional CA125 in postmenopausal patients [26]. A prospective study by this group further examined serum levels of CA125-sTn and CA125-MGL at the time of diagnosis, during treatment, and follow-up in HGSOC patients and found that CA125-sTn is a considerable indicator of tumor load and disease relapse [27]. In our previous study, the level of the pre-treatment serum Tn glycoform of CA125 (CA125-Tn) was elevated in ovarian cancer patients. CA125-Tn improves the differential diagnosis of ovarian cancer from ovarian borderline tumors and benign conditions such as endometriosis, adenomyoma, and pelvic infection with a higher specificity than conventional CA125 [28].

In this study, to further identify the clinical relevance of CA125-Tn as a serum biomarker for disease assessment, we analyzed the association of pre-treatment CA125-Tn levels with clinical factors in HGSOC. Moreover, we analyzed the potential of CA125-Tn in the preoperative assessment of peritoneal dissemination and surgery completeness and the prediction of disease recurrence in advanced HGSOC.

## Materials and methods

### Study populations

A total of 72 patients with a pathological diagnosis of HGSOC at the Obstetrics and Gynecology Hospital of Fudan University between November 2007 and September 2018 were retrospectively included in the study. The inclusion criteria were high-grade serous ovarian or fallopian cancer with serum CA125 ≥ 35U/mL before surgery and chemotherapy. Patients with malignancies in other organs and those who underwent palliative or fertility-sparing surgery were excluded. This study was approved by the Ethics Committee of the Obstetrics and Gynecology Hospital of Fudan University.

### Serum CA125-Tn detection

The remaining serum specimens were collected after CA125 testing in the clinical laboratory. CA125-Tn was examined using the CA125-Tn antibody-lectin enzyme-linked immunosorbent assay (ELISA) established previously [28]. Briefly, 100 µl of serum specimens was added to a 96-well microplate coated with the CA125 antibody. The microplate was then incubated with biotin-lectins and streptavidin-horseradish peroxidase (HRP) sequentially. Tetramethylbenzidine was used as a chromogenic substrate for HRP detection. The microplate was read at 405 nm using an ELISA reader. The value of CA125-Tn was calculated by subtracting A_405_ of the blank well from A_405_ of the sample well.

### Data collection

Patient characteristics were retrospectively collected from medical records, including age, menopausal status, pretreatment serum CA125 level, operative reports, surgical outcome, FIGO stage, and histological grade and type. Disease recurrence and progression-free survival (PFS) were also observed in stage III–IV cases. The tumor load of peritoneal dissemination in stage III–IV cases was evaluated using the quantitative model developed by Fagotti [29]. The total predictive index value (PIV) was calculated using parameters, including omental cake, peritoneal carcinosis, diaphragmatic carcinosis, mesenteric retraction, bowel and/or stomach infiltration, and liver metastases in the model. Each parameter was assigned a score of 2. The distribution patterns of peritoneal dissemination were evaluated using the criteria defined by Mayo Clinic, including three categories: pelvic disease, lower abdominal disease, and upper abdominal or miliary disease [7]. The parameters of peritoneal dissemination were obtained from operative reports.

### Statistical analysis

The normality of the CA125-Tn and CA125 levels was evaluated using the Shapiro–Wilk test, and the values were calculated as median, 25th, and 75th quartiles. Comparisons of CA125-Tn levels between clinicopathological subgroups were performed using the Kruskal–Wallis test. The association between CA125-Tn level and surgical completeness was assessed using univariate and multivariate logistic regression analyses. The predictive performance of CA125-Tn levels for surgical completeness was evaluated using receiver operating characteristic (ROC) curves. The optimal cutoff values were determined using the Euclidean index method. For prognostic analyses, CA125-Tn was analyzed as a dichotomous variable, with the cut-off value determined using X-tile version 3.6.1. PFS was evaluated using Kaplan–Meier curves and compared using the log-rank test. The associations of PFS with prognostic features were determined using univariate and multivariate Cox proportional hazards models. Statistical significance was set at *P* < 0.05. Statistical analyses were performed using the Stata version 15.1.

## Results

### Patient characteristics

A total of 72 patients with pretreatment serum CA125 ≥ 35U/mL and pathologically confirmed HGSOC were included in the analysis. Baseline patient characteristics are shown in Table 1. The mean age of the patients at surgery was 55.7 years. Forty-eight patients (66.7%) were postmenopausal women. The distribution of FIGO stage was stage I in seven patients (9.7%), stage II in six patients (8.3%), stage III in 54 patients (75.0%), and stage IV in five patients (6.9%). Comprehensive staging surgeries were performed in 13 early-stage patients (18.1%), while PDS and neoadjuvant chemotherapy (NACT)-interval debulking surgery (IDS) were performed in 51 patients (70.8%) and 8 patients (11.1%), respectively. Malignant ascites were found in 29 patients (55.8%). Immunohistochemical staining of CA125 in tumor tissues was reported in 40 patients, and 39 cases exhibited positive expression. Nineteen patients (26.4%) had endometriosis or adenomyosis.

**Table 1:** Baseline characteristics among 72 HGSOC patients in association with the pre-treatment serum CA125-Tn level

Characteristic	n (%)	CA125-Tn Median (IQR)	CA125 Median (IQR)
Age at surgery (years), mean (SD)	55.7 (9.6)	0.327 (0.190-0.614)	490.4 (180.3-1220.5)
Menopausal status			
Premenopausal	24 (33.3%)	0.326 (0.193-0.610)	756.9 (320.4-1655.5)
Postmenopausal	48 (66.7%)	0.327 (0.190-0.638)	373.9 (174.0-867.0)
*P*-value		0.830	0.126
FIGO stage			
I	7 (9.7%)	0.203 (0.063-0.267)	144.0 (119.5-931.8)
II	6 (8.3%)	0.213 (0.090-0.312)	140.9 (108.4-473.2)
III	54 (75.0%)	0.370 (0.207-0.691)	595.2 (276.8-1290.0)
IV	5 (6.9%)	0.337 (0.250-0.915)	269.0 (159.8-2944.0)
*P*-value		0.029	0.048
Treatment type			
Comprehensive staging surgery	13 (18.1%)	0.203 (0.070-0.290)	144.0 (119.5-473.2)
PDS	51 (70.8%)	0.337 (0.197-0.615)	590.3 (258.0-1387.0)
IDS	8 (11.1%)	0.803 (0.493-1.619)	542.8 (264.6-780.2)
*P*-value		0.0004	0.020
Malignant ascites			
Yes	29 (55.8%)	0.366 (0.220-0.769)	618.7 (179.2-1312.0)
No	23 (44.2%)	0.359 (0.182-0.606)	390.7 (167.9-931.8)
*P*-value		0.423	0.289
CA125-positive cells via immunohistochemistry			
<25%	20 (51.3%)	0.260 (0.200-0.556)	527.8 (156.0-1314.4)
25%-50%	8 (20.5%)	0.401 (0.224-0.560)	875.5 (371.2-1342.0)
>50%	11 (28.2%)	0.606 (0.317-0.769)	390.7 (207.7-2362.0)
*P*-value		0.115	0.814
Comorbidity with endometriosis / adenomyosis			
Yes	19 (26.4%)	0.290 (0.182-0.464)	390.7 (144.0-931.8)
No	53 (73.6%)	0.341 (0.197-0.615)	590.3 (207.7-1312.0)
*P*-value		0.511	0.174

*HGSOC* high-grade serous ovarian cancer, *IQR* interquartile range, *SD* standard deviation, *PDS* primary debulking surgery, *IDS* interval debulking surgery

### Association between pre-treatment serum CA125-Tn levels and clinicopathological characteristics

The association between pre-treatment serum CA125-Tn levels and clinicopathological characteristics was analyzed in all 72 HGSOC patients (Table 1). The median value of CA125-Tn was 0.327 (interquartile range [IQR]: 0.190–0.614) in all cases, and the median value of CA125 was 490.4 U/mL (IQR: 180.3–1220.5 U/mL). Elevated serum CA125-Tn levels were significantly associated with an advanced FIGO stage (*P* = 0.029). The level of CA125-Tn increased as the expression intensity of CA125 in tumor tissues increased, although no statistically significant difference was found (*P* = 0.115). There was no significant association between CA125-Tn and menopausal status, malignant ascites, or comorbidity with endometriosis or adenomyosis.

### Association between pre-treatment serum CA125-Tn levels and peritoneal dissemination in advanced HGSOC

To analyze the potential of pre-treatment serum CA125-Tn to reflect peritoneal dissemination, the tumor load and distribution patterns of peritoneal dissemination were retrospectively evaluated in 59 advanced (FIGO stage III-IV) HGSOC patients according to the operative reports. PIV and distribution patterns provide a direct anatomical view of the peritoneal dissemination. A high tumor load with PIV ≥ 8 is a significant predictor of unresectable disease and major post-operative complications [30]. The features of peritoneal dissemination among the 59 patients with advanced HGSOC are summarized in Table 2. The median PIV was 6 (range: 0–10), and 14 patients (23.7%) had PIV ≥ 8. The level of CA125-Tn increased along with the elevation of the PIV score (*P* = 0.004) and was significantly higher in the patients with a PIV ≥ 8 than in those with PIV < 8 (*P* = 0.0001). Pre-treatment serum CA125 levels showed similar changes among the PIV score groups.

**Table 2:** Features of peritoneal dissemination and residual disease among 59 advanced HGSOC patients in association with the pre-treatment serum CA125-Tn level

Parameter	n (%)	CA125-Tn Median (IQR)	CA125 Median (IQR)
PIV			
0	9 (15.3%)	0.202 (0.157-0.262)	507.5 (137.9-976.7)
2	10 (16.9%)	0.268 (0.169-0.615)	344.2 (181.4-1387.0)
4	8 (13.6%)	0.313 (0.227-0.551)	255.2 (179.5-367.1)
6	18 (30.5%)	0.339 (0.207-0.691)	706.8 (443.5-910.0)
8	11 (18.6%)	0.915 (0.411-1.309)	1659.0 (375.4-2944.0)
10	3 (5.1%)	0.926 (0.461-1.248)	2693.0 (573.6-5000.0)
*P*-value		0.004	0.039
PIV category			
PIV <8	45 (76.3%)	0.315 (0.182-0.594)	507.5 (197.3-910.0)
PIV ≥8	14 (23.7%)	0.919 (0.461-1.248)	2010.5 (466.8-2944.0)
*P*-value		0.0001	0.005
Peritoneal dissemination pattern			
Pelvic	3 (5.1%)	0.182 (0.096-0.536)	507.5 (137.9-1151.0)
Lower abdominal	16 (27.1%)	0.235 (0.163-0.382)	350.6 (175.1-1013.4)
Upper abdominal or miliary	40 (67.8%)	0.479 (0.316-0.916)	634.5 (297.7-1655.5)
*P*-value		0.011	0.234
Residual disease			
0	17 (28.8%)	0.220 (0.157-0.411)	328.8 (168.8-976.7)
≤1cm	22 (37.3%)	0.350 (0.207-0.606)	627.3 (258.0-1652.0)
>1cm	20 (33.9%)	0.694 (0.355-1.001)	634.5 (373.9-2176.0)
*P*-value		0.005	0.129

*HGSOC* high-grade serous ovarian cancer, *IQR* interquartile range, *PIV* predictive index value

As shown in Table 2, three patients (5.1%) were categorized as having pelvic disease, 16 patients (27.1%) had lower abdominal disease, and 40 patients (67.8%) had upper abdominal or miliary disease. Upper abdominal and miliary diseases were combined into one pattern for analysis because of the common co-existence of these two spread patterns in patients [7]. The difference in CA125-Tn levels was significant in three categories: miliary or upper abdominal > lower abdominal > pelvic disease (*P* = 0.011), whereas the difference in CA125 levels showed no statistical significance among peritoneal dissemination patterns (*P* = 0.234).

### Prediction of pre-treatment serum CA125-Tn levels in surgical completeness in advanced HGSOC

To evaluate the prediction of pre-treatment serum CA125-Tn levels in surgical completeness, the largest residual tumor diameter was retrospectively collected in 59 advanced HGSOC patients according to the operative reports. As summarized in Table 2, RD = 0, RD ≤ 1 cm, and RD > 1 cm were left in 17 (28.8%), 22 (37.3%), and 20 (33.9%) patients, respectively, after debulking surgery. The level of CA125-Tn increased with RD diameter (*P* = 0.005), whereas no statistically significant difference in CA125 levels was found among the three groups (*P* = 0.129). Moreover, the association between CA125-Tn level and suboptimal surgery (RD > 1 cm) remained significant (adjusted odds ratio 5.78, *P* = 0.029) even after adjusting for treatment type (PDS versus IDS) and FIGO stage (III vs. IV) (Table 3). These data indicate that pre-treatment serum CA125-Tn level might be a suitable predictor of surgical completeness.

**Table 3:** Univariable and multivariable analysis of the pre-treatment serum CA125-Tn level for suboptimal surgery

Variable	Univariable		Multivariable	
	OR (95% CI)	*P*-value	OR (95% CI)	*P*-value
CA125-Tn	6.680 (1.475-30.254)	0.014	5.781 (1.196-27.927)	0.029
FIGO stage (III vs. IV)	1.333 (0.204-8.708)	0.764	1.292 (0.170-9.819)	0.804
Treatment type (PDS vs. IDS)	4.000 (0.847-18.901)	0.080	1.765 (0.294-10.600)	0.534

*OR* odds ratio, *CI* confidence interval, *PDS* primary debulking surgery, *IDS* interval debulking surgery

**Fig. 1:**
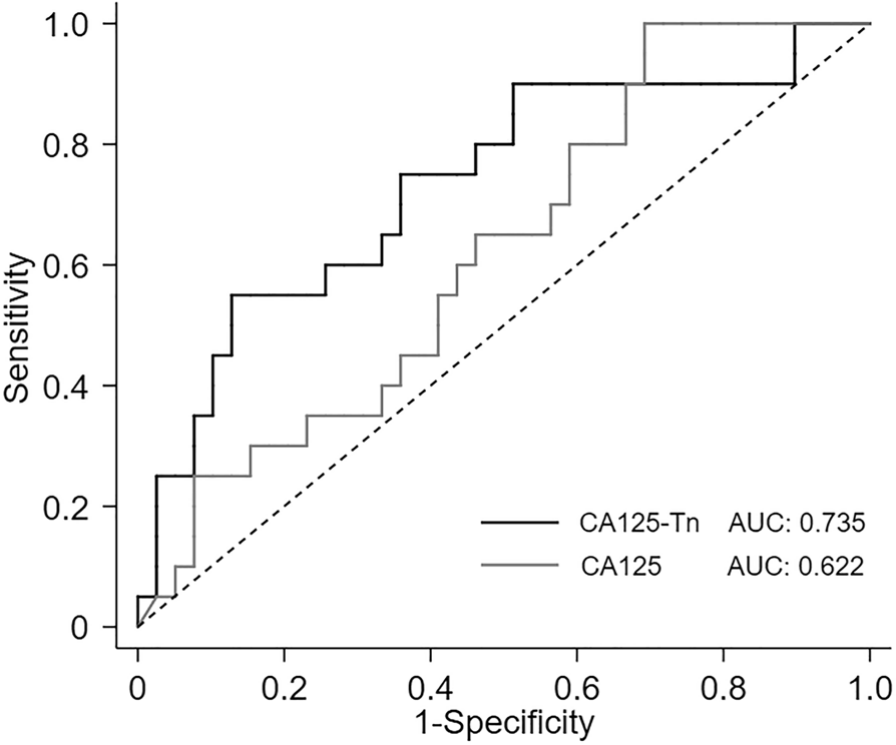
The ROC analysis of the pre-treatment serum CA125-Tn and CA125 to discriminate suboptimal surgery in advanced HGSOC patients. ROC: receiver operating characteristic; HGSOC: high-grade serous ovarian cancer

The discriminating performance of pre-treatment serum CA125-Tn levels for suboptimal surgery was assessed using ROC analysis, in which the area under the curve (AUC) was 0.735 (Fig. 1). In contrast, the AUC of the pre-treatment serum CA125 level was 0.622. The optimal cut-off values, sensitivity, specificity, and classification accuracy are listed in Table 4. The sensitivity, specificity, and classification accuracy with the CA125-Tn cut-off level were 75.0%, 64.1%, and 67.8%, respectively, for suboptimal surgery prediction, which was stronger than the discriminating performance of pre-treatment CA125.

**Table 4:** The discriminating performance of the pre-treatment serum CA125-Tn for suboptimal surgery

Parameter	Cut-off value	Sensitivity (%)	Specificity (%)	Classification accuracy (%)
CA125-Tn	0.373	75.0	64.1	67.8
CA125 (U/mL)	573.6	65.0	53.9	57.6

### Association between pre-treatment serum CA125-Tn levels and prognosis in advanced HGSOC

**Fig. 2:**
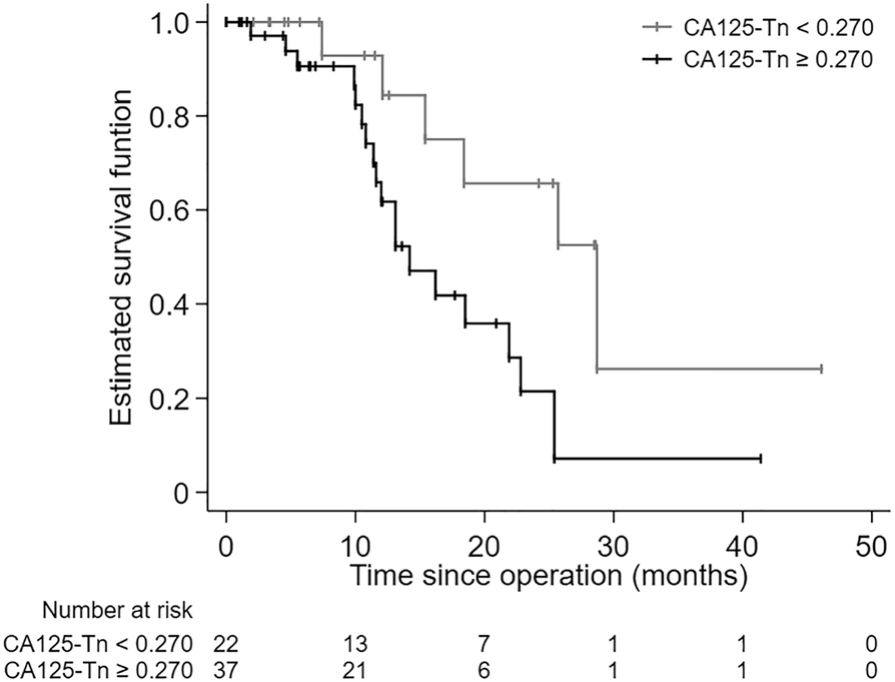
Kaplan–Meier curves for PFS in advanced HGSOC patients. PFS arranged by the pre-treatment serum CA125-Tn. The Kaplan–Meier method with log-rank test was used for the analysis of survival difference. The cut-off value of CA125-Tn was determined by X-tile software. PFS: progression-free survival; HGSOC: high-grade serous ovarian cancer

To evaluate the potential of serum CA125-Tn levels in the prognosis of advanced HGSOC, PFS was analyzed in this study. The follow-up duration ranged from 1 to 73.1 months. A total of 25 patients (42.4%) developed disease recurrence during the follow-up period. Kaplan–Meier curves for PFS of patients with pretreatment CA125-Tn cut-off values are illustrated in Fig. 2. The PFS was significantly longer for patients with CA125-Tn levels < 0.270 (median: 28.7 months) than for patients with CA125-Tn levels ≥ 0.270 (median: 14.2 months) (log-rank test, *P* = 0.014). The crude and adjusted hazard ratios (HRs) are shown in Table 5, using univariable and multivariable Cox proportional hazards models. In the univariate analysis, pretreatment CA125-Tn ≥ 0.270 was significantly associated with PFS (HR = 3.092, 95% confidence interval [CI]: 1.199–7.973). After adjustment for age, FIGO stage (III vs. IV), and RD, the HR of pre-treatment CA125-Tn ≥ 0.270 was 2.728 (95% CI: 1.002–7.429) for disease recurrence. Baseline pretreatment CA125-Tn levels might not independently predict disease recurrence.

**Table 5:** Univariable and multivariable Cox proportional hazards models for PFS.

Variable	Univariable		Multivariable	
	HR (95% CI)	*P*-value	HR (95% CI)	*P*-value
CA125-Tn ≥ 0.270	3.092 (1.199–7.973)	0.020	2.728 (1.002–7.429)	0.050
Age	0.973 (0.924–1.025)	0.304	0.972 (0.924–1.022)	0.270
FIGO stage (III vs. IV)	1.814 (0.536–6.137)	0.338	1.822 (0.486–6.837)	0.374
Residual disease > 1 cm	2.203 (0.988–4.915)	0.054	2.006 (0.839–4.799)	0.118

*PFS *progression-free survival, *HR *hazard ratio, *CI *confidence interval

## Discussion

In this study, we demonstrated for the first time the association between the CA125-Tn glycoform and clinical factors in HGSOC. High levels of pre-treatment serum CA125-Tn were found in advanced FIGO stages and indicated higher tumor load and more aggressive patterns of peritoneal dissemination. Moreover, the pre-treatment CA125-Tn level increased in the presence of RD and could serve as a predictor of surgical completeness in HGSOC. Although pre-treatment CA125-Tn was not identified as an independent risk factor for disease recurrence, a high level indicated a shorter PFS in patients with advanced HGSOC.

Abnormal glycosylation of CA125 promotes tumor–mesothelial adhesion by providing binding sites for ovarian cancer cells to interact with multiple adhesion molecules on the mesothelial cell surface [31, 32]. Compared with normal cells, most cancer cells exhibit an immature truncated O-glycophenotype and show accumulation of Tn and sTn antigens, which correlates with poor prognosis [33, 34]. The level of CA125-sTn is elevated in 44% of ovarian cancer patients, but not in all patients with endometriosis, and increases as both stage and grade advance in ovarian cancer [35]. The high level of CA125-sTn at diagnosis correlates with a high tumor load in the peritoneal cavity of HGSOC patients [27]. Similarly, we recently reported that the pre-treatment serum CA125-Tn level was significantly elevated in ovarian cancer compared with borderline ovarian tumors and benign conditions [28]. The current study further identified that the pre-treatment serum CA125-Tn level increased as the tumor stage and tumor burden of peritoneal dissemination advanced in HGSOC patients. In particular, CA125-Tn levels increased with the aggressiveness of the peritoneal dissemination pattern, whereas CA125 levels showed no similar changes in advanced HGSOC patients. High levels of CA125-Tn in HGSOC patients with miliary and upper abdominal disease, the most neglected or occult metastasis type, might be valuable for disease management. The strong association between CA125-Tn and peritoneal dissemination also provides inspiration for other peritoneal tumors. Future studies should uncover the potential underlying mechanisms between CA125-Tn and peritoneal metastasis.

Peritoneal metastasis is the major cause of suboptimal cytoreduction, and 60% of patients experience peritoneal relapse, even with complete remission after primary surgery [36, 37]. Evaluation of peritoneal dissemination is necessary to predict surgical outcomes. In advanced ovarian cancer, the R0 rate has been reported as only 8.1–33.5% [38]. However, the resection rate is often overestimated because RD is commonly assessed by surgeon inspection and palpation. Patients with complete PDS may have radiological residual disease on postoperative CT scans [39]. RD lesions > 1 cm can still be detected through postoperative CT scans in approximately 40% of patients undergoing optimal cytoreduction [4].

Preoperative assessment of surgical outcomes using a minimally invasive approach is instructive for treatment choice. If millimetric RD or significant surgery comorbidities are predicted after PDS, NACT-IDS may be superior due to similar PFS, less complex surgery, and fewer perioperative complications [30, 40]. A few preoperative and intraoperative predictive models for complete or optimal cytoreduction have been developed for advanced ovarian cancer, including clinical factors, CA125 levels, radiological examination, laparoscopy, and laparotomy-based evaluation [29, 41–45]. The valid tumor tissue-based biomarkers predicting postoperative RD have not been identified [46]. Molecular subtypes from postoperative tumor mRNA profiling can improve the predictive performance of preoperative CT for surgical resectability in patients with advanced HGSOC in a pilot study [47].

Therefore, a non-invasive and convenient serum biomarker remains to be identified. Preoperative serum CA125 level has been investigated as a predictive factor and is included in several predictive models. A CA-125 cutoff of ≥ 500 U/mL was used to predict suboptimal cytoreduction and ≥ 600 U/mL was used to predict any RD in the clinico-radiological models from Sudian et al. [41, 42]. A CA-125 level ≥ 500 U/mL showed a sensitivity of 78% and specificity of 73% for predicting suboptimal cytoreduction in stage III ovarian cancer [48]. In a model based on preoperative CT scans and clinical factors, a CA125 level > 800 U/ml was a significant clinical factor for suboptimal cytoreduction in advanced ovarian cancer [49]. Even a CA125 level > 1000 U/ml is included in a laparoscopic risk-adjusted model to predict postoperative outcomes from Fagotti et al. [30].

However, the prediction performance of pre-operative CA125 levels for the surgery outcome of cytoreduction still remains controversial, with a low sensitivity and specificity [50, 51]. Some chronic medical conditions besides ovarian cancer influence CA125 levels, in turn weakening the specificity or sensitivity of CA125 in ovarian cancer detection [52–54]. We recently reported elevated specificity of CA125-Tn in differentiating ovarian cancer from borderline and benign ovarian tumors [28]. In the current study, patients with higher CA125-Tn levels might be less likely to undergo R0 resection, and a higher CA125-Tn level might indicate a larger RD diameter. CA125-Tn exhibited better predictive performance than CA125 for the surgical outcome of cytoreduction, with higher sensitivity, specificity, and accuracy. However, pre-treatment CA125-Tn level might not be an independent predictor of prognosis, although a high level indicated a shorter PFS in patients with advanced HGSOC. The prognostic value of CA125-Tn should be evaluated in prospective studies and in the context of other clinical features.

Our study had some limitations. Due to the retrospective study design, we did not observe dynamic changes in CA125-Tn levels in patients with HGSOC after treatment. The value of CA125-Tn for reflecting disease outcome requires further verification in longitudinal analysis. Another limitation is that we could not collect data on the peritoneal cancer index (PCI) for peritoneal dissemination evaluation owing to the retrospective design. PCI can quantitatively provide a more direct anatomical view of the extent of peritoneal dissemination.

## Conclusion

In conclusion, pretreatment serum CA125-Tn levels were related to tumor staging, peritoneal dissemination, and RD. CA125-Tn could be a novel biomarker for peritoneal dissemination and a promising predictor of surgical completeness in ovarian cancer that could help surgeons adopt optimized treatment strategies for patients with advanced ovarian cancer as a pre-treatment evaluator. Patients with lower CA125-Tn levels were more likely to have no RD. Further research is needed to validate the clinical value of CA125-Tn in disease management and to explore the biological mechanisms of CA125-Tn and disease progression.

## Data Availability

The datasets used and analyzed in the current study are available from the corresponding author upon reasonable request.
